# Humanized mouse model reveals the immunogenicity of Hepatitis B Virus vaccine candidates produced in CRISPR/Cas9-edited *Nicotiana benthamiana*

**DOI:** 10.3389/fimmu.2025.1479689

**Published:** 2025-04-09

**Authors:** Iuliana Caras, Irina-Elena Ionescu, Ana-Maria Pantazica, André van Eerde, Hege Steen, Inger Heldal, Sissel Haugslien, Catalin Tucureanu, Raluca-Elena Chelmus, Vlad-Constantin Tofan, Adriana Costache, Adrian Onu, Hang Su, Norica Branza-Nichita, Jihong Liu-Clarke, Crina Stavaru

**Affiliations:** ^1^ Research and Development Department, “Cantacuzino” Institute, Bucharest, Romania; ^2^ Department of Viral Glycoproteins, Institute of Biochemistry of the Romanian Academy, Bucharest, Romania; ^3^ Division of Environment and Natural Resources, Norwegian Institute for Bioeconomy Research (NIBIO), Ås, Norway

**Keywords:** HBV, humanized mice, virus-neutralization, chimeric antigen, CRISPR/Cas9-edited *Nicotiana benthamiana*, immune response

## Abstract

**Introduction:**

Hepatitis B Virus (HBV) infection is still an ongoing public health issue worldwide. The most efficient tool in preventing HBV infection remains vaccination and significant efforts have been made in the last decade to improve current HBV vaccines. Owing to the strict HBV tropism for the human liver, developing animal models for preclinical screening of vaccine candidates is extremely challenging. To date, there are only a few reports regarding the use of humanized mouse models for the evaluation of the immunogenic properties of viral antigens.

**Methods:**

Previously we showed that a *Nicotiana benthamiana*-produced HBV-S/preS1^16-42^ antigen elicited strong HBV-specific immune responses in BALB/c mice. In the current study, we used immunodeficient NOD.Cg-Prkdc^scid^ Il2rg^tm1Wjl^/SzJ (NSG) mice as recipients of human peripheral blood mononuclear cells (hPBMCs), to evaluate the immunogenicity of the recently developed chimeric HBV immunogen produced in CRISPR/Cas9-edited *N. benthamiana*, under more “humanized” conditions.

**Results:**

Analysis of the immune response in NSG mice immunized with the chimeric antigen demonstrated induction of virus infection-neutralizing antibodies, indicating activation of antigen-specific B cells.

**Discussion:**

The ability of hPBMCs-engrafted NSG mice to mount specific humoral immune responses after immunization with viral antigens supports this animal model as a promising tool for pre-clinical evaluation of human vaccine antigens.

## 1 Introduction

**Figure 1:**
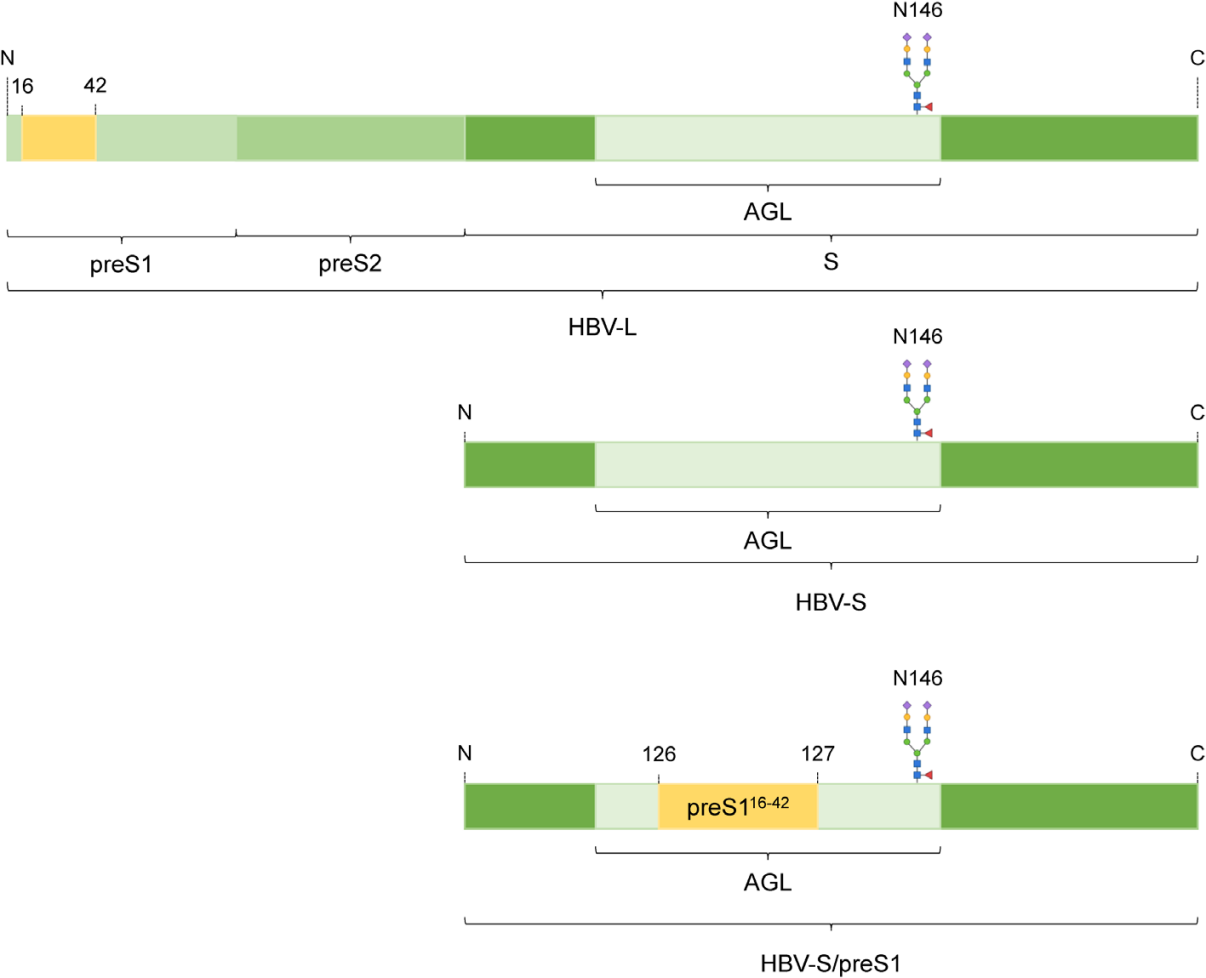
Schematic representation of the HBV-S/preS1^16-42^ antigen. The HBV envelope proteins large (HBV-L) and small (HBV-S) derive from the same open reading frame and share the S-domain. The 16-42 amino acid sequence from the preS1 domain of the L protein was introduced into the antigenic loop (AGL) of the S protein, between amino acids 126 and 127 to result the HBV-S/preS1^16-42^ antigen. The N-glycan attached at position 146 is shown.

Worldwide, more than 800,000 people die every year from Hepatitis B Virus (HBV)- related causes. Chronic HBV infections are currently treated with modulators of the immune response and replication inhibitors that must be taken for life. Thus, immunization remains the most efficient measure to control HBV infection and commercial vaccines based on expression of the HBV small (S) envelope protein in yeast are used in universal or selective vaccination programs (1). However, issues associated with lack of response, decrease in long-term protection in vaccinated individuals and emergence of variants with vaccine-escape mutations (VEM) that are not recognized by the anti-S antibodies elicited by the current vaccine have highlighted a need for more immunogenic HBV antigens, incorporating the large (L) and medium (M) envelope proteins (2, 3). We have recently developed a novel HBV chimeric vaccine candidate by inserting L-derived preS1 sequences into the major antigenic loop (AGL) of the S protein (**Figure 1**). This approach ensures equimolar display of immunologically relevant -S and -L epitopes, avoiding the need to produce the full-length L protein that is notoriously difficult to express (4, 5). Our previous study showed that the S/preS1^16-42^ antigen induced enhanced immunogenicity when compared to the S protein as well as neutralizing antibodies against both wild type (WT) and VEM HBV variants (5). To mitigate costs associated with production in mammalian cells, we also investigated alternative production systems, namely plants with a “humanized” N-glycosylation (FX-KO) system (6). The results of our previous study showed that immunization with S/preS1^16-42^ obtained in FX-KO *N. benthamiana* induced significantly higher antibody titers in mice, with more potent neutralizing activity against both native and VEM-HBV, as compared with the antigen produced in WT plants. This indicated that FX-KO *N. benthamiana* is a reliable platform for cost-efficient production of improved HBV antigens (7). Evaluation of novel vaccine candidates in the appropriate immune system context is crucial for efficient screening of viral antigens at preclinical level.

Mouse models have significantly advanced our understanding of human immunology (8); however, their application in investigating the immunogenicity of new vaccines has been challenging. Despite shared physiological and metabolic processes, the evolutionary gap between mice and humans results in substantial variations in how each species mobilizes its immune system against diseases (9), which include variations in immune cell composition, spatiotemporal expression of immune proteins and receptors, hematopoiesis, and cytokine function (9). Such differences may explain the frequent disparities observed in the efficacy of novel vaccines and drugs between animal experiments and human trials.

Non-human primates are a potential option to represent the effect of novel vaccines and therapeutics on humans more accurately. However, ethical concerns and high costs associated with experiments on non-human primates highlight the need for alternative animal models that successfully combine the advantages of each model while lessening their limitations (10).

Thus, significant effort has been made to develop mouse models with “humanized” immune systems that could be used as a cost-effective system to study human immune responses to disease, as well as new vaccines and therapeutic compounds. In the past decades, advancements in our understanding of the molecular mechanisms governing innate and adaptive immunity have resulted in the development of mouse models that possess increased capability to incorporate human cells and tissues (11).

The common cytokine receptor γ chain (γc, encoded at the locus Il2rg) is a receptor subunit shared by interleukin 2 (IL-2), IL-4, IL-7, IL-9, IL-15, and IL-21 receptors. Mutations in this protein are responsible for diseases characterized by the absence of T cells and NK cells (12). Surprisingly, studies showed that mice exhibiting different Il2rg-based immunodeficiencies had robust development of human hematopoietic stem cells after transplantation (13). As such, the introduction of Il2rg-based immunodeficient mouse models significantly advanced the understanding of human infectious diseases, cancer development and autoimmune diseases, by enabling robust development of human T cells and effective activation of both cell-mediated and humoral immunity (14). These improved models overcome limitations seen in earlier severe combined immunodeficiency disease (SCID) mice, providing valuable tools for studying human immune responses.

In this work we used a recently developed chimeric HBV immunogen produced in CRISPR/Cas9-edited *N. benthamiana* plants (7), as a model antigen to investigate the suitability of NOD.Cg-Prkdc^scid^ Il2rg^tm1Wjl^/SzJ (NSG) mice transplanted with human peripheral blood mononuclear cells (hPBMCs) for evaluation of vaccine candidates in the context of a “humanized” immune response.

The novel HBV antigen, namely HBV-S/preS1^16-42^, combines relevant epitopes from the S and large (L, preS1 domain) envelope proteins (4, 5), inducing a superior immune response in BALB/c mice when compared to the S protein (5). Our results show that hPBMCs-engrafted NSG mice immunized with the novel HBV antigen are able to mount specific humoral immune response with significant virus infection- neutralization activity against both WT and a VEM-HBV variant.

To the best of our knowledge, this is the first evaluation of an HBV vaccine candidate in an animal model with a “humanized” immune response.

## 2 Materials and methods

### 2.1 Cell lines, viruses and plasmids

HEK293T and Huh7 cell lines were cultured in Dulbecco’s Modified Medium (DMEM) supplemented with 10% fetal bovine serum (FBS) and 1% non-essential amino acids (NEAA). For HBV neutralization assays, HepG2hNTCP cells (provided by Professor Stephan Urban, German Center for Infection Research, University of Heidelberg) were cultured as previously described (5). HepG2.2.2.15 cells, which are transfected stably with two copies of the HBV genome (gift from Dr. David Durantel, INSERM U871, Lyon, France), were utilized for WT HBV stock production, as reported earlier (15). All cell lines were maintained at 37°C with 5% CO_2_ in an incubator with a humidified atmosphere. Stocks of HBV with a clinically relevant VEM, namely the G145R mutation within the antigenic domain of the S protein, were obtained by transfecting Huh7 cells with pGEM-4Z-HBV 1.3 G145R (5) using Lipofectamine 3000 (Invitrogen) according to the manufacturer’s protocols. Cell media were collected between days 7 and 12 post-transfection, concentrated via ultracentrifugation on a 20% sucrose layer. The viral stocks were then further purified to eliminate subviral particles (SVPs) via sucrose gradient ultracentrifugation as described in Wettengel et al. (16). Fractions containing HBV and lacking SVPs were determined via real-time qPCR and ELISA, respectively, and then quantified using real-time qPCR to determine viral titers (17).

### 2.2 Production of HBV-S/preS1^16-42^ antigen in CRISPR/Cas9 double knock-out FX-KO *Nicotiana benthamiana* lines

The HBV-S/preS1^16-42^ antigen was produced in CRISPR/Cas9-edited *N. benthamiana* lines with knockouts in their α (1, 3)-fucosyltransferase and β (1, 2)-xylosyltransferase genes (FX-KO) through vacuum-based agroinfiltration, as previously described (7). The S/preS1^16-42^ antigen- encoding gene was introduced into the expression plasmid vector pEAQ-ht-DEST1 using Gateway cloning as described in a previous publication (6). The S/preS1^16-42^ plasmid vector was transformed into Agrobacterium tumefaciens LBA4404 via electroporation. Plant leaves were infiltrated with Agrobacterium suspension and subsequently harvested 7 or 8 days after infiltration (7).

### 2.3 Antigen purification from the CRISPR/Cas9 double knockout FX-KO *Nicotiana benthamiana* plants

Leaves harvested from FX-KO *N. benthamiana* plants expressing the S/preS1^16-42^ antigen were ground in liquid nitrogen and homogenized in 5 volumes of lysis buffer (0.15 M NaCl, 20 mM Na2HPO4, 20 mM sodium ascorbate, 0.1% Triton X-100, pH 7) containing a cocktail of protease inhibitors (Santa Cruz Biotechnology), as previously described (7).

Lysates were then concentrated via ultracentrifugation on a 20% sucrose layer, for 5 h at 30,000 rpm (SW 32 Ti, Beckman Coulter) and then further separated via ultracentrifugation on a 15-60% sucrose gradient at 32,000 rpm (SW 42Ti, Beckman Coulter) for 18 h to isolate high-molecular weight SVPs. Collected fractions were tested for the presence of HBV antigens via ELISA using the Monolisa HBsAg Ultra kit (BioRad). Positive fractions were then pooled, dialyzed against PBS and incubated with 1% activated carbon (Carbocit^®^, S.C. Biofarm S.A.) for 3 h at 4°C with head-over-tail agitation, to remove smaller protein impurities, and then further purified via size-exclusion chromatography on the CaptoCore 400 resin (Cytiva), by gravitational flow, as previously described (7). Flow-through fractions containing S/preS1^16-42^ SVPs were identified via ELISA as described above, pooled and dialyzed against PBS 0.01X and then SpeedVac-concentrated at 35°C for 5 h (Thermo Scientific-Pierce). The antigen was then quantified by western blot and Coomassie staining under denaturing conditions, as previously described (4).

### 2.4 SDS-PAGE and western-blot analysis

Protein samples were heat-denatured by boiling for 10 min in Laemmli buffer and then were separated via sodium dodecyl sulfate 12% polyacrylamide gel electrophoresis (SDS-PAGE) and subjected to either Coomassie staining (using ReadyBlue Protein Gel Stain from Sigma-Aldrich) or transferred onto nitrocellulose membranes. After blocking the membranes in 10% skimmed dry milk in PBS for 1 h, the membranes were incubated with mouse anti-preS1 antibodies (sc-57762, Santa Cruz Biotechnology, 1:1,000) overnight at 4°C, followed by a 1 h incubation at room temperature (RT) with HRP-conjugated mouse-IgGκ binding protein (sc-516102, Santa Cruz Biotechnology, 1:10,000). Protein visualization was accomplished using an enhanced chemiluminescence assay (ECL, Thermo Scientific-Pierce). Protein densitometry analysis was performed using Image J software from the National Institutes of Health, and antigen concentrations were estimated through a standard curve generated with commercial L protein processed under the same experimental conditions (preS1 antigen, Beacle).

### 2.5 Humanized mice engraftment and immunization protocol

Female NSG mice (stock number 005557) were purchased from Jackson Laboratory (Bar Harbor, ME). All animals were housed in specific pathogen-free (SPF) conditions and used at the age of 8-10 weeks. Animal experiments were conducted in accordance with national and institutional guidelines for animal care and were approved by the Institute Animal Ethics Committee and by the National Authority ANSVSA nr. 488/22.01.2020.

hPBMCs were obtained from the peripheral venous blood of three healthy volunteers after obtaining informed consent. The cells were isolated via Ficoll-Hypaque density gradient centrifugation (1.077 g/mL density, Merck, Darmstadt, Germany). The isolated hPBMCs, suspended in PBS, were injected at 1×10^7^ cells/dose, intraperitoneally (ip) into NSG mice.

Groups of 5-7 NSG mice were reconstituted with hPBMCs of each donor depending on the available cell number. The success of the transplant was demonstrated by human lymphocytes phenotyping via flow-cytometry using mice peripheral blood collected on the fourth day after transplantation. hPBMCs-engrafted mice were immunized by intramuscular injection with 30 µg/dose of HBV-S/preS1^16-42^ antigen purified from FX-KO *N. benthamiana* adjuvanted with Addavax (InVivoGen) (n=11) or with Addavax only as control (n=8). The immunization protocol consisted of a prime immunization, the fifth day after hPBMCs engraftment, followed by two boosters at 2-week intervals. The mice were monitored daily and their body weight was verified twice per week. One week after the last boost (day 35), spleens and serum samples were collected (**Table 1**). Mice showing clinical deterioration before the last boost were removed from the study. Serum samples were processed for detection of human antibodies by ELISA and neutralization analysis, and spleen cells were used for evaluation of cellular immune response by Luminex multiplex assays and FACS analysis.

**Table 1:** Flow chart of the experimental protocol for human cells engraftment and the immunization protocol.

Day	NSG mice
-5	1×10^7^ PBMCs per mouse
-1	Peripheral blood collection • Flow cytometry analysis
0	Immunization
14	
28	
35	Serum and spleen harvest • Immune response evaluation

### 2.6 Immunophenotyping of peripheral blood and splenocytes in transplanted mice by flow-cytometry

Peripheral blood and spleens were obtained from hPBMCs-engrafted mice and were prepared as single-cell suspensions. Human immune cell populations were detected using mAbs specific for the following human antigens: CD3-PE (clone HIT3), CD14-FITC (clone HCD14), and CD19-APC (clone 4G7). Isotype antibodies were used as negative controls and reported values were corrected using these isotype control values.

To exclude murine host cells, anti-mouse Ly-6G/Ly-6C Pacific Blue (Gr-1) (clone RB6-8C5) monoclonal antibody staining was performed in all experiments. All antibodies were purchased from BioLegend (San Diego, CA). Specific mAbs were added to the samples and incubated for 30 min at 4°C. Stained peripheral blood samples were then washed and red blood cells were lysed using BD FACS Lysing solution (BD Biosciences) followed by analysis via flow-cytometry. At least 50 000 events were acquired on the FACSCanto II instrument (BD Biosciences). Data analysis was performed using the BD FACSDiva Software v.6.1.2.

### 2.7 Analysis of the humoral immune response in vaccinated hPBMCs-engrafted mice

Blood samples were collected under anesthesia from individual mice by orbital puncture at the end of the experiment. Blood sera were obtained from clotted blood following centrifugation at 14,000 × g for 10 min at RT then stored at -80°C until further use for antibody titration. The antigen-specific human IgG (hIgG) and IgM (hIgM) were detected via ELISA as previously described (5), by using a suspension of UV-inactivated HBV in PBS (containing 0.4 μg/mL of HBsAg) for plate coating and anti-human IgG H&L (HRP) (ab6858) or anti-human IgM mu chain (HRP) (ab97205), as secondary antibodies. Statistical analysis was performed by using the Wilcoxon rank-sum test.

### 2.8 Analysis of the cellular immune response in vaccinated mice

Splenocytes isolated from immunized mice were seeded in 96-well culture plates at 0.5×10^6^ and 0.1×10^6^ cells/well in RPMI-1640 media containing 10% fetal calf serum (FCS) and 2-mercaptoethanol (50 µM, Sigma-Aldrich). Triplicate samples were stimulated with UV-inactivated HBV (10 µg/mL HBsAg). Unstimulated cells were used as control samples for the baseline response and cell functionality was assessed by stimulation with Concanavalin A (ConA 5 µg/mL, Sigma-Aldrich). The cultures were incubated for 36 h at 37°C, 5% CO_2_. The supernatant was then removed and stored at -80°C for later use. Cellular immune responses were analyzed by detecting cytokine secretion using a human Luminex-based bead multiplex assay (LXSAHS-14, R&D Systems) according to the manufacturer’s instructions. We selected a panel of 14 cytokines secreted by different subclasses of immune cells (IP-10, IL-23, MIP-1α, IFN-γ, IL-2, IL-10, IL-17, TNF-α, MCP-1, IL-1β, IL-4, IL-6, IL-13, IL-5). Statistical analysis was performed by using the Wilcoxon rank-sum test.

### 2.9 HBV infection neutralization assay

HepG2hNTCP cells were plated in 48-well plates one day before HBV infection. Sera collected from immunized mice at day 35 post-immunization were diluted 1:20 in complete DMEM media with 4% polyethylene glycol (PEG, Sigma Aldrich) and incubated for 1 h at 37°C with WT HBV and the VEM HBV-SG145R (100 genome equivalents/cell). The pre-inoculum was then used to infect the plated HepG2hNTCP cells. Control conditions included cell treatment with 1 µM Myrcludex B (Pepscan), a well-established HBV entry inhibitor (18), for 3 h before virus inoculation, as well as cells infected with untreated HBV. At 16 h post-infection, cells were thoroughly washed with PBS and incubated with complete DMEM media supplemented with 2.5% DMSO (AppliChem). The medium collected between days 7-11 post-infection was used to quantify HBsAg secretion through the Monolisa HBs Ag-Ab PLUS kit (BioRad). The inhibition of HBV infection by the immune sera was expressed as a percentage relative to infectivity values in the presence of pre-immune sera at the same dilution. Statistical analysis was conducted using the Mann-Whitney U test in GraphPad Prism version 6.

## 3 Results

### 3.1 Production and purification of HBV-S/preS1^16-42^ antigen from FX-KO *N. benthamiana* plants

**Figure 2:**
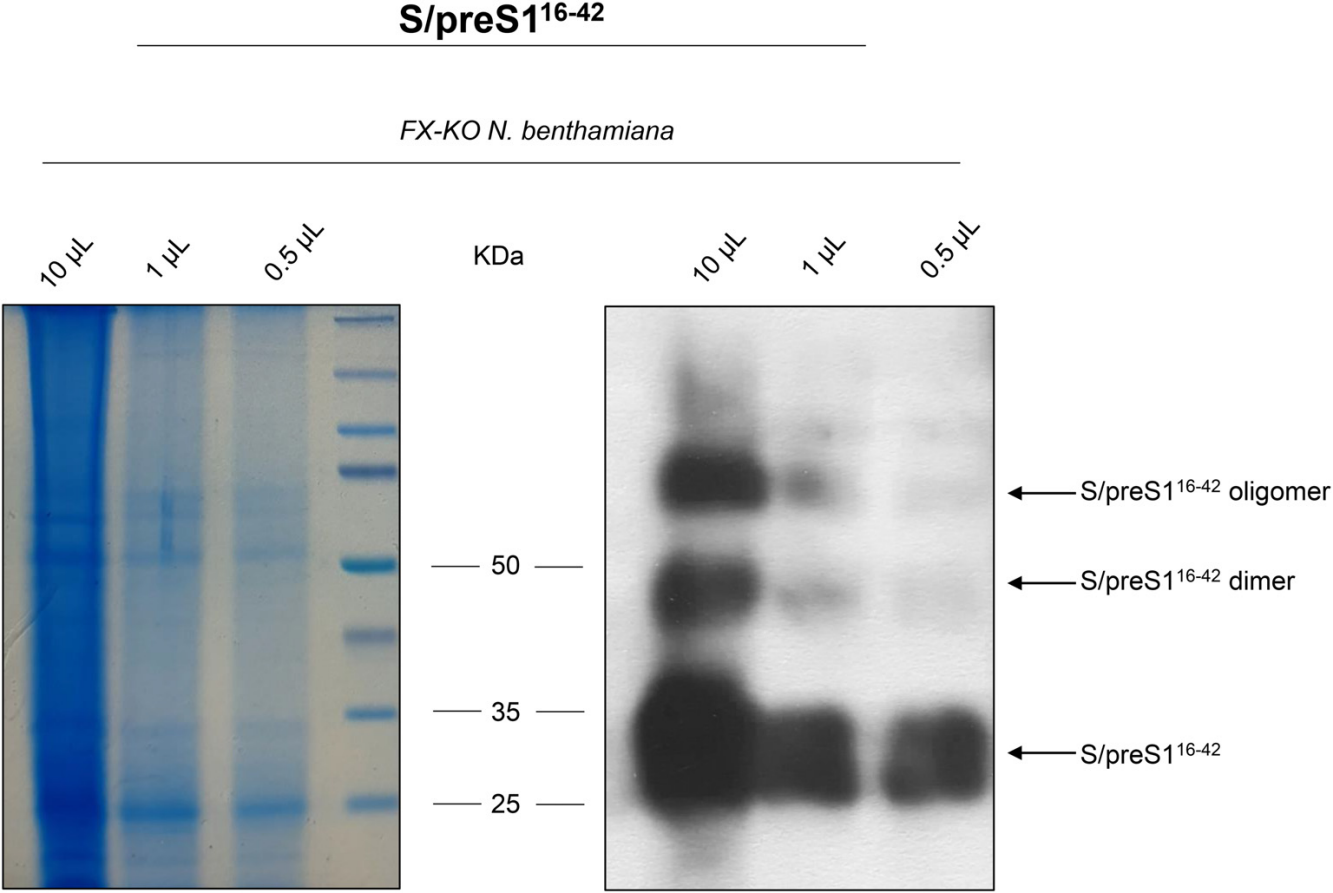
Analysis of HBV-S/preS1^16-42^ antigen produced in FX-KO *N. benthamiana* plants. Lysates from FX-KO *N. benthamiana* leaves expressing HBV-S/preS1^16-42^ antigen were 20-fold concentrated followed by fractionation using a 15-60% sucrose gradient to isolate high-molecular weight SVPs. Antigen-positive fractions were dialyzed against PBS, incubated with activated carbon and then subjected to gel filtration. Samples were concentrated and analyzed via Coomassie staining (left panel) and western blot (right panel) under reducing conditions using anti-preS1 antibodies.

The S/preS1^16-42^ chimeric antigen (**Figure 1**) was produced by transient expression in CRISPR/Cas9-edited *N. benthamiana* plants with a humanized N-glycosylation pathway (FX-KO) (7). To obtain sufficient quantities of antigen for the immunization experiments, 50 g of FX-KO *N. benthamiana* leaves expressing the S/preS1^16-42^ antigen were lysed and the resulting extracts were concentrated via 20% sucrose-bed ultracentrifugation to isolate assembled SVPs. SVPs were further purified via sucrose gradient ultracentrifugation and gel filtration of antigen-positive fractions on CaptoCore 400 as previously described (7). Samples were concentrated and analyzed by SDS-PAGE followed by either Coomassie staining, or western blot and detection with anti-preS1 antibodies (**Figure 2**). These analyses revealed the presence of a major, broad band at ~25 kDa, representing the monomeric antigen, and of weaker, higher molecular weight bands corresponding to protein dimers and oligomers. Total protein and S/preS1^16-42^ quantification indicated an antigen purity of ~80%.

### 3.2 Immunophenotyping of peripheral blood demonstrates successful engraftment of hPBMCs in NSG mice

**Figure 3:**
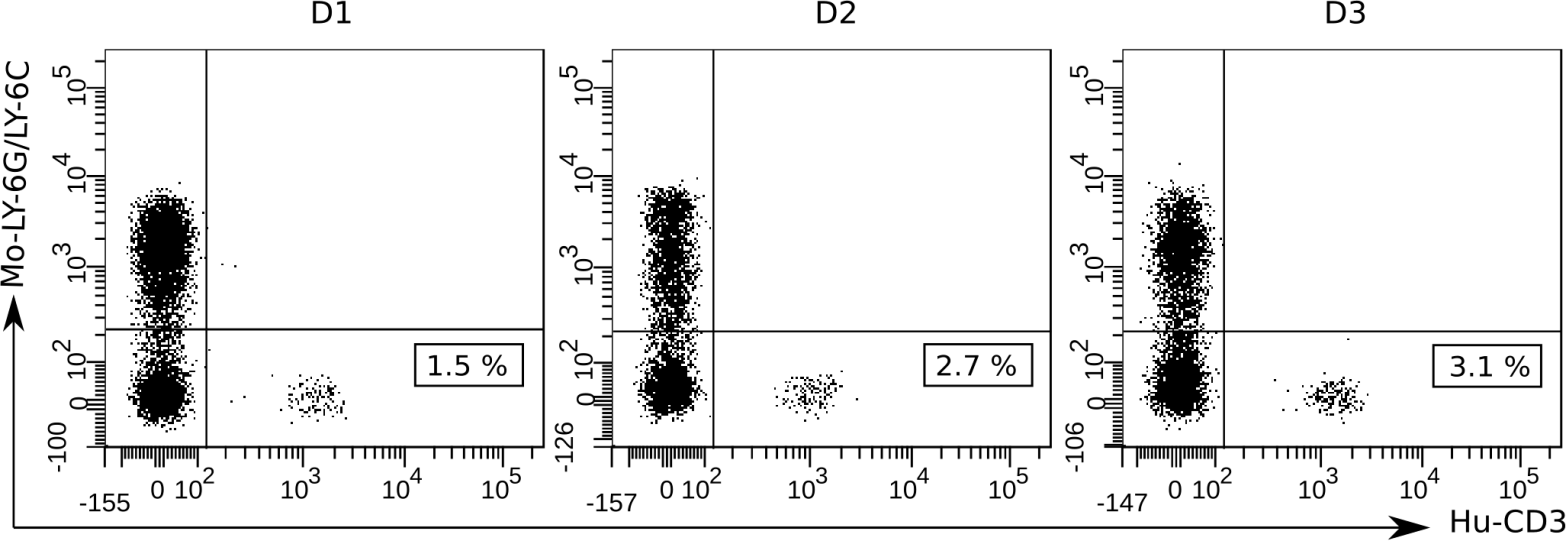
Flow-cytometric detection of hPBMCs engraftment in the blood of NSG mice at 4 days after human cell transplantation (day -1). To detect human T cells and exclude murine host cells, a gating strategy involving anti-mouse Ly-6G/Ly-6C Pacific Blue and anti-human CD3-PE mono-clonal antibody staining was used. At least 50 000 events were acquired and analyzed using the BD FACSDiva Software v.6.1.2. Data shown are representative dot plots depicting the gating strategy for three individual mice transplanted with hPBMCs from the three different donors (D1, D2, and D3). Murine Ly-6G/Ly-6C+ cell population (Mo-Ly-6G/Ly-6C) is represented in the upper left quadrants and human CD3+ cell population (Hu-CD3) and the cell percentages are represented in the bottom right quadrants.

To verify hPBMCs engraftment, we analyzed human leukocytes in the peripheral blood of recipient mice 4 days after intraperitoneal injection of 1×10^7^ hPBMCs (day -1). A gating strategy using anti-mouse Ly-6G/Ly-6C staining was employed to exclude murine cells. As shown in **Figure 3**, human CD3+ T cells were detected in all mice, with percentages ranging from 1 to 3%. However, human CD19+ B cells and CD14+ monocytes were not detected by flow-cytometry analysis in transplanted mice. A table detailing CD19+ and CD14+ cell percentages is shown in the supplementary (**Supplementary Table S1**). These results suggest that at least part of the hPBMCs were successfully engrafted into NSG mice.

### 3.3 Immunophenotyping of splenocytes demonstrates the presence of a mixed population of human immune cells in transplanted mice

**Figure 4:**
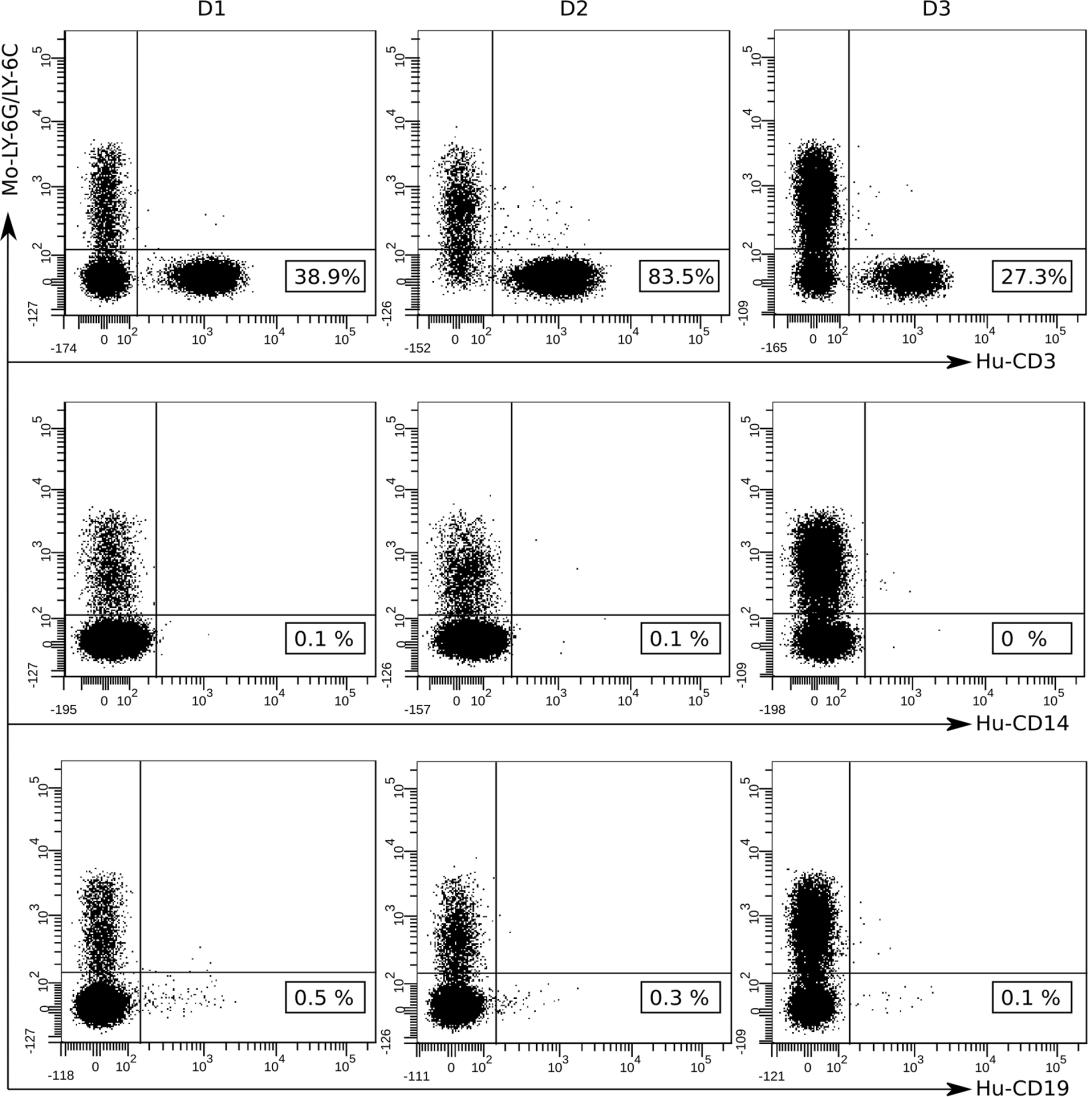
Flow-cytometry analysis of hPBMCs detected in the spleens of NSG mice at 35 days after first immunization. The presence of human mononuclear cells in the spleen of engrafted mice was detected via immunophenotyping analysis after staining with fluorochrome labeled anti-human antibodies: CD3-PE, CD14-FITC, CD19-APC and anti-mouse Ly-6G/Ly-6C Pacific Blue. At least 50 000 events were acquired and data were analyzed using the BD FACSDiva Software v.6.1.2. Data shown are representative dot plots depicting the gating strategy for three individual mice transplanted with hPBMCs from the three different donors (D1, D2, and D3). Murine Ly-6G/Ly-6C+ cell population (Mo-Ly-6G/Ly-6C) is represented in the upper left quadrants and human CD3+ (Hu-CD3), CD14+ (Hu-CD14) and CD19+ (Hu-CD19) cell populations and the cell percentages are represented in the bottom right quadrants.

Secondary lymphoid tissues, including the spleen, are sites of antigen presentation and lymphocyte activation and are consequently critical for antigen-specific immune response evaluation. Therefore, we analyzed the presence of human CD3+ T cells in the spleen of hPBMCs-engrafted mice on day 35 after first immunization. The same gating strategy was applied for splenocytes as for peripheral blood cells to exclude murine cells. Immunophenotyping analysis revealed high engraftment rates of human CD3+ T cells in the spleen of humanized mice while human B cells and monocytes were barely detected (**Figure 4**).

### 3.4 Analysis of the humoral immune response shows the presence of hIgG and hIgM in transplanted mice following immunization with the S/preS1^16-42^ antigen

**Figure 5:**
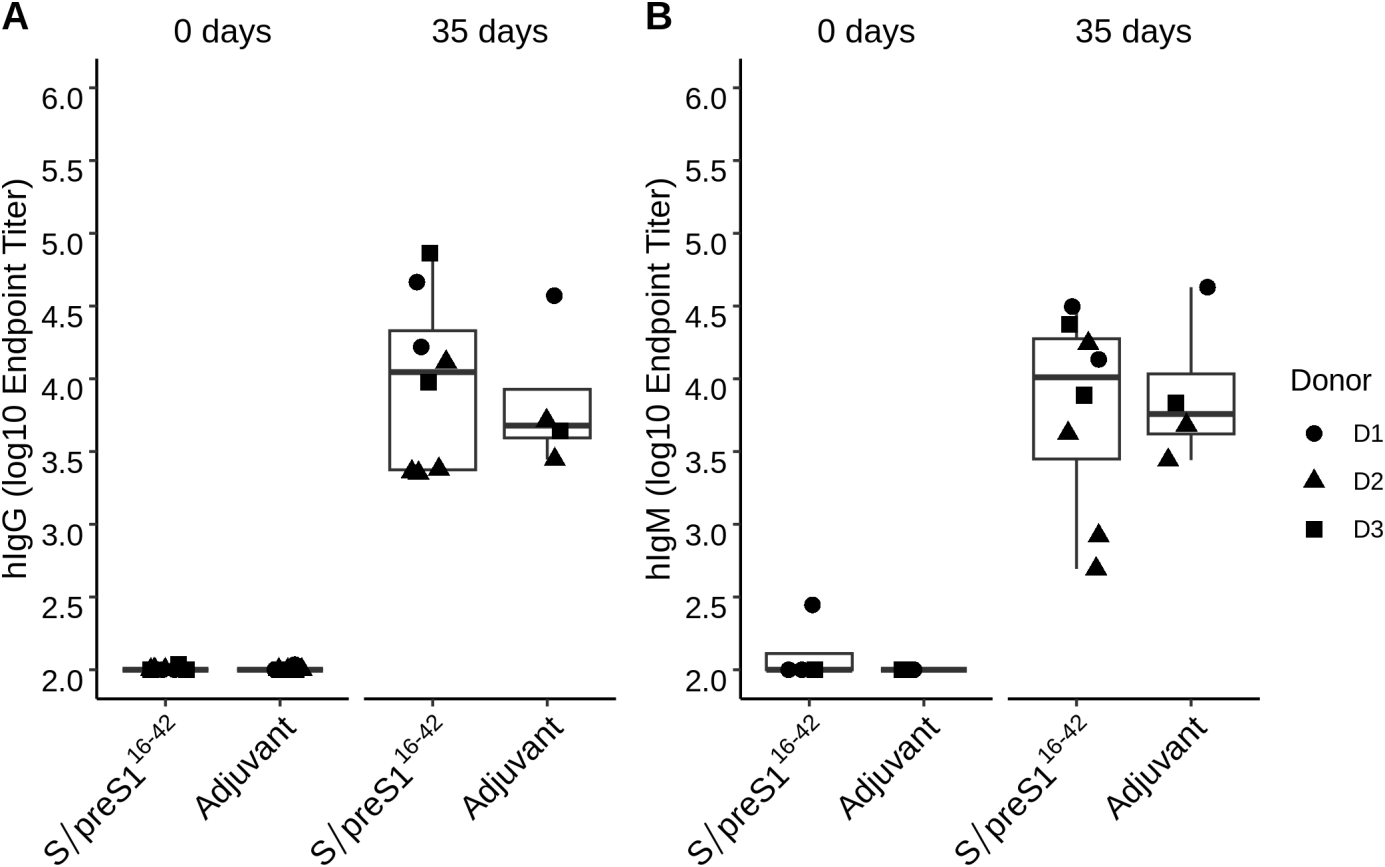
Analysis of the humoral immune response indicates the presence of hIgG and hIgM in transplanted mice following immunization with the S/preS1^16-42^ antigen. Groups of mice engrafted with hPBMCs from three donors (D1, D2, and D3) were immunized 3 times at 2-week intervals with Addavax-adjuvanted FX-KO *N. benthamiana*-produced S/preS1^16-42^ antigen (S/preS1^16-42^) or adjuvant alone (Adjuvant). hIgG **(A)** and hIgM **(B)** endpoint titers at 0- and 35-days post-immunization were calculated based on a 4-parameter logistic regression curve fitted to a pool of immune sera, as the reciprocal sample dilution that would result in three times baseline ± standard error as derived from the internal standard curve.

The humoral immune response of NSG mice was investigated following three immunizations with the S/preS1^16-42^ antigen administered at 14-day intervals. As clinical deterioration was observed in these mice, serum samples were collected one week after the last boost. The presence of antigen-specific hIgG and hIgM was determined by ELISA assay using plates coated with inactivated HBV as previously described (5). At 35 days post-immunization elevated hIgG and hIgM titers were detected in both the adjuvant control group (Addavax) and the S/preS1^16-42^ antigen group. Higher hIgG and hIgM responses were observed in the antigen immunization group (**Figure 5**). Due to the inherent heterogeneity of the humoral immune response previously observed in BALB/c mice (7), the genetic variability between the human donors and the small number of mice per group, this response was not statistically significant (**Figure 5**). The hIgG and hIgM endpoint titers for each individual mouse are presented in the supplementary section (**Supplementary Table S2**). The lack of significant differences between the two groups may also be explained by the properties of the used adjuvant Addavax, which is a squalene-based oil-in-water nano-emulsion that can activate the immune response in a nonspecific way (19).

### 3.5 Analysis of the cellular immune response reveals the presence of human cytokines in transplanted mice following immunization with the S/preS1^16-42^ antigen

**Figure 6:**
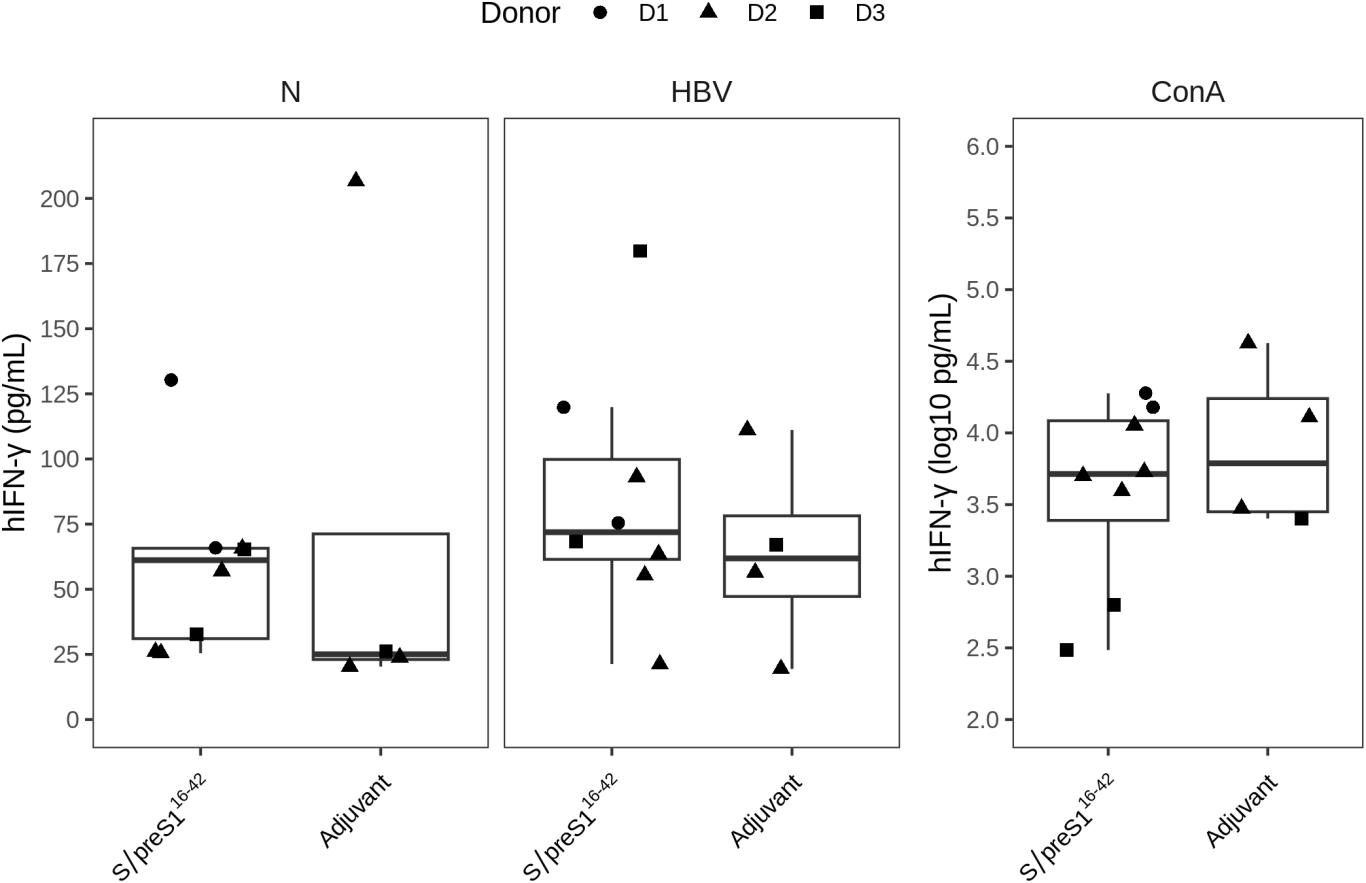
The cellular immune response triggered by FX-KO *N. benthamiana*-produced S/preS1^16-42^ antigen (S/preS1^16-42^). Groups of mice engrafted with hPBMCs from three donors (D1, D2, and D3) were immunized with Addavax-adjuvanted FX-KO *N. benthamiana*-produced S/preS1^16-42^ antigen (S/preS1^16-42^) or adjuvant alone (Adjuvant). The level of human IFN‐γ cytokine (hIFN‐γ) secreted by spleen cells harvested at day 35 was detected using the luminex multiplex assay after *in vitro* stimulation with UV‐inactivated HBV (HBV) or PBS (control cells, N). Stimulation with concanavalin A (ConA) was used as a positive control for cell survival and functionality. The hIFN‐γ secretion levels (pg/mL) are shown as boxplots, and the ConA stimulation secretion levels are shown separately in a logarithmic scale (log10 pg/mL) to better illustrate the magnitude of the response.

To evaluate antigen-specific cellular immune response of hPBMCs-transplanted NSG mice, isolated splenocytes were stimulated ex-vivo for 36 h with UV-inactivated HBV and secretion of various human cytokines was measured in cell culture supernatants. We found that antigen stimulation induced only a slight increase in IFN-γ levels in HBV-S/preS1^16-42^ antigen-immunized groups compared to adjuvant-control groups with no statistical difference between the two immunization groups or as compared with the unstimulated cells (**Figure 6**). Notably, from the 14 cytokines tested, except IFN-γ, all other tested cytokines had minimal to negligible secretion levels. However, the functional activity of the isolated splenocytes was highlighted by *in vitro* cytokine secretion in response to polyclonal ConA stimulation, which was used as a positive control (data not shown).

### 3.6 Immunization with the S/preS1^16-42^ antigen in NSG mice induces human antibodies with potent virus-neutralizing activity against WT-HBV and VEM-HBV

**Figure 7:**
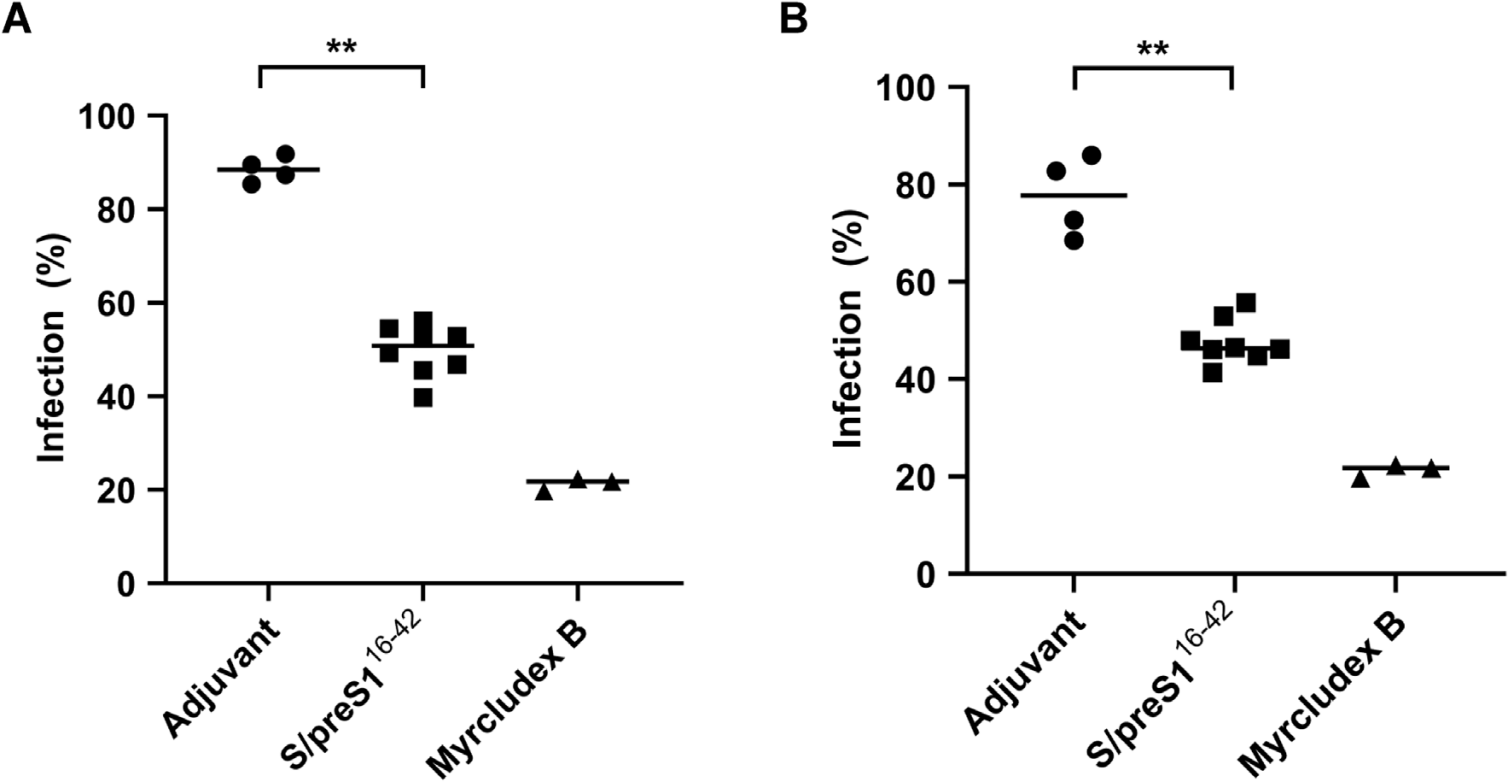
WT-HBV **(A)** and VEM-HBV **(B)** neutralization activity of immune sera from NSG mice immunized with FX-KO *N. benthamiana*-derived S/preS1^16-42^ antigen. Sera from mice transplanted with hPBMCs and immunized with Addavax-adjuvanted FX-KO *N. benthamiana*-produced S/preS1^16-42^ antigen (S/preS1^16-42^) or adjuvant alone were diluted 1:20 and pre-incubated with WT- or VEM-HBV inoculum (100 Geq/cell) for 1 h and then used to infect HepG2hNTCP cells for 16 h Cells incubated with Myrcludex B for 3 h prior to infection were used as a negative control. Cell media were collected at day 11 post-infection and infection levels were quantified by determining HBeAg levels via ELISA. Data are shown as percentage of HBV infection in the presence of post-immune sera from infection values obtained in the presence of the pre-immune sera, at the same dilution, with each datapoint representing an animal. Values in the presence of Myrcludex B represent percentages of infection from HBV-only samples. Data from two independent experiments run in triplicate (animal sera) or duplicate (controls) biological samples are shown. Horizontal bars indicate the median values within each group. Statistical analysis was performed by using the Mann-Whitney U test. **, p < 0.01.

Finally, to more accurately determine whether immunization with the S/preS1^16-42^ antigen in NSG mice could give rise to a neutralizing antibody response, we evaluated the capacity of sera to protect from HBV infection *in vitro*. WT-HBV and a vaccine-escape variant containing the SG145R mutation were obtained as previously described (5) and purified. HepG2hNTCP cells were then infected with an inoculum containing virus (100 Geq/cell) and pre- or post-immune sera (1:20 dilution) from NSG mice vaccinated with antigen or adjuvant. Pre-treatment with Myrcludex B was used as a positive control of specific inhibition of viral entry. As shown in **Figure 7** a strong neutralization ability of the immune sera from NSG mice immunized with the S/preS1^16-42^ antigen (of about 50%) was observed against both WT-HBV (**Figure 7A**) and VEM-HBV (**Figure 7B**), when compared to the group immunized with only adjuvant. This suggests that the antibodies induced by S/preS1^16-42^ immunization in NSG mice are specific and potently neutralizing, which is consistent with the immunogenicity profile shown by this antigen in other mouse models (5). Notably, we have previously observed a higher HBV neutralization activity of ~ 75%, for sera from BALB/c mice immunized with the same FX-KO *N. benthamiana* – derived S/preS1^16-42^ antigen (7). However, this effect is likely due to the significantly higher IgG titers triggered by vaccination in BALB/c mice, as compared to the NSG animal model.

## 4 Discussion

Since HBV was identified in the 1960s, vaccines against HBV infection containing different antigens produced in various expression platforms have been reported over several decades. HBV antigen produced in plants can be traced back to the 1990s (20), and now our previous studies have reported that HBV antigen was successfully produced in WT and CRISPR/Cas9-edited *N. benthamiana* and showed immunogenicity BALB/c in a mouse model, enhancing an efficient cellular and humoral immune response (5, 7). In addition, red alga Porphyridium produced hepatitis C virus (HCV) glycoprotein showing an ability to elicit a specific immune response in the BALB/c mouse model (21). In the present study, we investigated the feasibility of hPBMCs-humanized mice to evaluate the novel HBV antigens produced in CRISPR/Cas9-edited *N. benthamiana* to induce immune responses in humans. This takes the plant produced vaccine a step forward by testing in a mouse model, which is more humanized, for future application against HBV, and also other viral infections such as HCV.

Humanized mouse models have been widely used in the last decade for the study of human infectious diseases, cancers, and autoimmune diseases and for *in vivo* validation of new therapeutic approaches, including drugs, vaccines, and immunotherapy, thereby emerging as a new gold standard model in translational research (22–24) despite limitations due to the presence of mouse MHC, differences in growth factors and cytokines required for immune system development, and deficiencies in the development and organization of lymph nodes (25). Humanized mouse models for HBV research have primarily focused on development of treatments for chronic HBV and understanding the complex immune response involved in HBV infection. Thus, there is little research regarding the use of mice with humanized immune responses for evaluation of novel vaccine candidates against HBV, with most studies focusing on using either classic mouse strains or non-primate models for pre-clinical HBV vaccine candidates (23).

The most common methods of engrafting immunodeficient mice with functional human cells include the transplantation of hPBMCs, CD34+ hematopoietic stem cells, and bone marrow-liver-thymus fetal tissue (26). Of these, hPBMCs engraftment is more commonly used, as they are easier to obtain and retain the immune memory of the donor cells post-engraftment, enabling the study of the immune response induced by vaccine-derived antigenic stimulation based on individual human immune response that is more relevant compared to classical murine models. However, this model offers a limited window for experimental use due to the development of xenogeneic graft-versus-host disease (GvHD)-like symptoms a few weeks after transplantation (27–29). The advantage of this model is the ease of obtaining and engrafting hPBMCs and the possibility to use growth factors and cytokines to increase survival, class switching and activity of B-cells (23). Similarly, the transfer of human hPBMCs or immune cells derived from human hPBMCs, such as donor-matched dendritic cells, can be optimized to increase the magnitude of specific immune response (30).

In this study, we investigated the feasibility of hPBMCs-humanized mice for the evaluation of the potential of novel HBV antigens to induce an immune response in humans. As a model antigen for immunization, we have chosen the newly developed chimeric S/preS1^16-42^ antigen produced in *N. benthamiana*, based on its ability to elicit specific humoral and cellular immune responses in standard mouse models (8). We first investigated successful transplantation of hPBMCs derived from volunteer donors into NSG mice. Previous studies have shown that most of the engrafted hPBMCs in NSG mice are represented by CD3+ T cells, while B cells and monocytes are present at very low or undetectable levels (31). Consistently, the results of flow-cytometry analysis after hPBMCs engraftment in the peripheral blood of NSG mice revealed the presence of human CD3+ cells, while human CD19+ B cells and CD14+ monocytes were not detected. The low percentage of human T cells detected in the peripheral blood four days after intraperitoneal injection of hPBMCs suggests a delayed entry into the bloodstream and retention in the peritoneal cavity. Our results are consistent with those of previous reports which show low levels of human CD3+ T cells during the first 2-3 weeks post-engraftment and a significant increase at 4 weeks after intraperitoneal injection of hPBMCs (32).

The proportion of human cells was also assessed in the spleen of “humanized mice” at the end point of the experiment. Flow-cytometry analysis showed that a high percentage of the spleen cells isolated from immunized hPBMCs-engrafted mice were human CD3+ T cells while human B cells and monocytes were barely detected, confirming that at least part of the hPBMCs were successfully engrafted. These results are consistent with previous studies showing a high number of human T cells while human B cells, NK cells, dendritic cells and monocytes were detected at low levels in the spleens of hPBMCs-NSG mice (32, 33). Another study of engraftment kinetics of hPBMCs in NSG mice before GVHD development revealed that the majority of the engrafted cells were T cells and only a small percentage were B cells. Moreover, while T-cells expanded, the proportion of B-cells contracted during the 4-week time course with distribution in peripheral blood, spleen, lymph nodes, and bone marrow (34).

To evaluate whether antigen-specific T-cell responses could be induced in hPBMCs-NSG mice, splenocytes isolated from immunized mice were stimulated *in vitro* with UV-inactivated HBV and specific cytokines were measured in cell culture supernatants. Antigen stimulation resulted in increased IFN-γ secretion in the S/preS1^16-42^ antigen group when compared with the adjuvant control group, although the differences observed were not significant. However, polyclonal ConA stimulation induced significant IFN-γ production, indicating that the engrafted cells were functional. These results could be due to the limited engraftment and survival of human antigen presenting cells and the difficulty of detecting antigen-specific activation of T cells due primarily to human T cell reactivity against mouse MHC molecules in this model (23).

Next, we evaluated whether antigen-specific B cell responses could be induced in the immunized mice. Although the number of B cells identified in the peripheral blood and spleen was relatively low, serum samples from immunized mice at 35 days post-immunization showed the presence of both hIgG and hIgM. Notably, these antibodies were also detected in mouse sera following adjuvant immunization. These results are in accordance with a previous report showing that hPBMCs engrafted into immunodeficient mice could provoke non-specific antibody responses within 2–4 weeks post-engraftment of hPBMCs, which may mask the antigen-specific response (35).

The essential property of an efficient humoral immune response to vaccination is the capacity to neutralize the pathogen infection. To assess the specificity of the humoral immune response induced by the S/preS1^16-42^ antigen, the HBV neutralization activity of the sera from immunized mice was measured. We observed a strong inhibition of HBV infection in the presence of immune sera from NSG mice immunized with the S/preS1^16-42^ antigen when compared to the group immunized with only adjuvant, which demonstrates that the B cell response detected is antigen-specific. Our results are in agreement with other studies that reported survival and IgG production by human B cells transferred into NSG mice (36) and induction of neutralizing antibodies against severe acute respiratory coronavirus by immunization of hPBMCs-engrafted NSG mice with cDNA constructs encoding the structural antigens of the virus (37). Concomitant detection of hIgM in vaccinated NSG mice is not surprising as it appears to be the predominant antibody response in other humanized mice models (28). Due to their pentameric structure enabling multivalent binding to the envelope proteins exposed on the viral surface, it is conceivable that hIgM antibodies contributed at least partly to the significant HBV neutralization activity of the immune sera.

Despite low frequencies of B cells in immunized mice, there are several possible explanations for the intriguing presence of HBV-neutralizing antibodies: (i) the activation and differentiation of B cells into short-lived plasma cells producing hIgM followed by low hIgG, seroconversion and/or (ii) migration of activated B cells to various lymphoid tissues where they survived and continued to produce human antibodies. Moreover, GvHD in PBMC-engrafted mice could affect B cell distribution and survival in lymphoid tissues.

## 5 Conclusion

In conclusion, our results showed that, despite a limited experimental window, the hPBMCs-engrafted NSG mice are able to mount specific humoral immune responses after immunization with viral antigens. However, the inherent heterogeneity of the immune response, together with the genetic variability of the human cell donors must be taken into account for potential use of this animal model in preclinical screening of human vaccine candidates. Our data also confirmed the immunogenicity of the S/preS1^16-42^ antigen produced in FX-KO *N. benthamiana* in the context of a “humanized” immune response, warranting further investigation of this antigen as a promising alternative for immunization of poor responders to the current S-based vaccine.

## Data Availability

The original contributions presented in the study are included in the article/**Supplementary Material**. Further inquiries can be directed to the corresponding authors.

## Supplementary material

Supplementary Figure 1 Full scan of the original SDS-PAGE gel.

Supplementary Figure 2 Full scan of the original Western blot scan.
