# Supplementary figures and images for: Humanized mouse model reveals the immunogenicity of Hepatitis B Virus vaccine candidates produced in CRISPR/Cas9-edited Nicotiana benthamiana
Source: Front Immunol. 2025 Apr 9;16:1479689. doi: 10.3389/fimmu.2025.1479689 (PMC12014679; doi:10.3389/fimmu.2025.1479689)

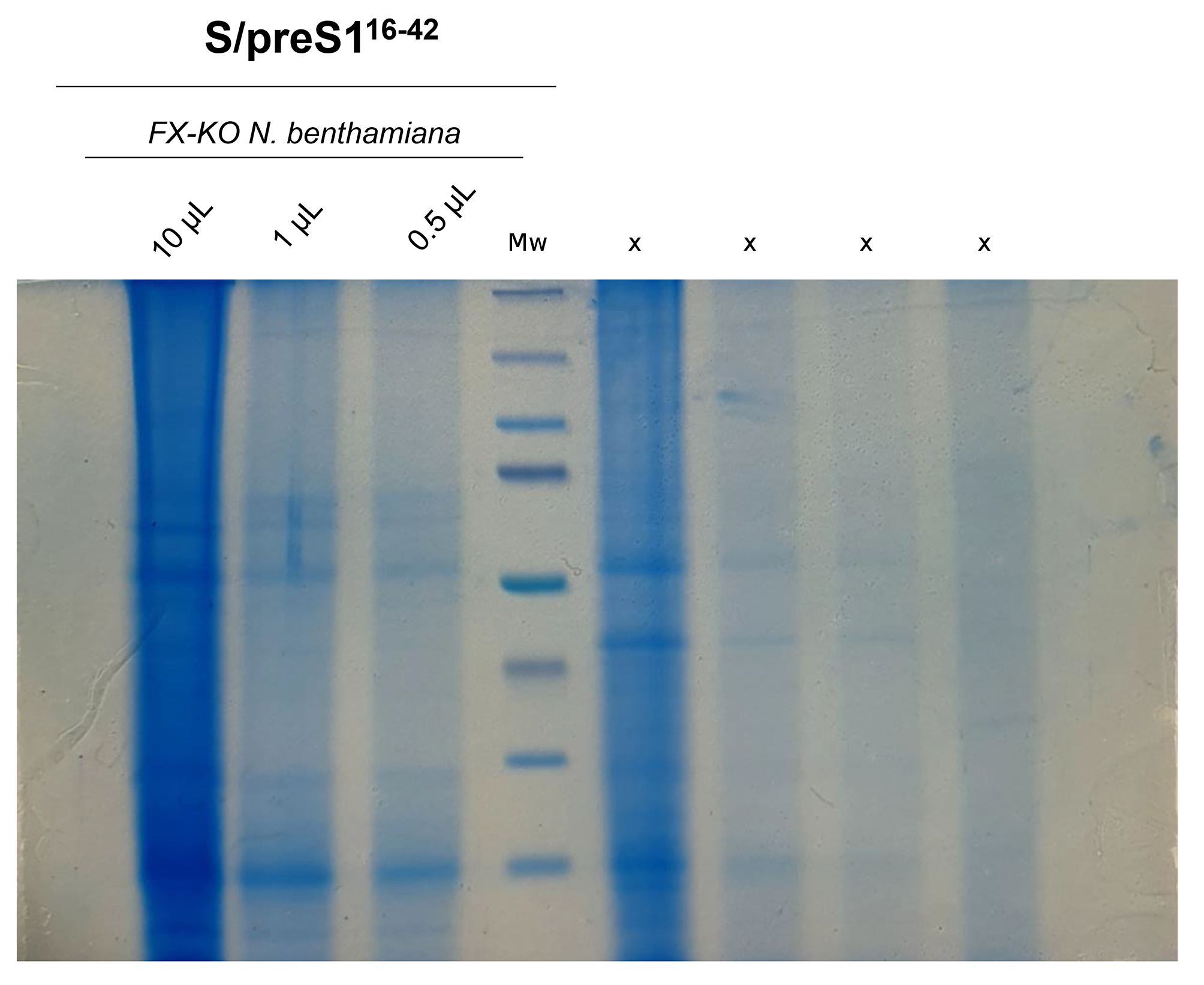

Supplement: Supplementary Figure 1 — Full scan of the original SDS-PAGE gel. [file Image1.tif]

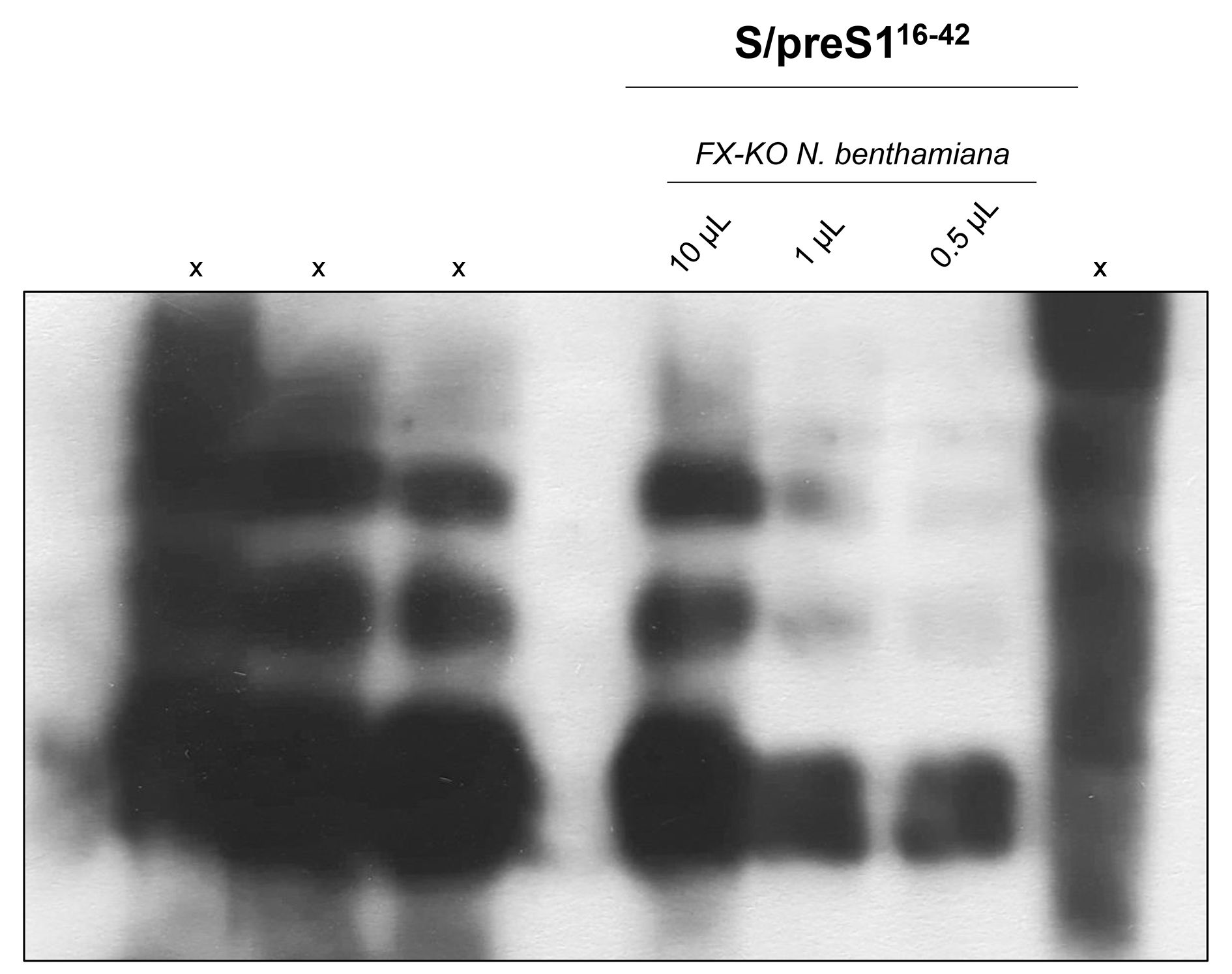

Supplement: Supplementary Figure 2 — Full scan of the original Western blot scan. [file Image2.tif]
