# Supplementary material for: Humanized mouse model reveals the immunogenicity of Hepatitis B Virus vaccine candidates produced in CRISPR/Cas9-edited Nicotiana benthamiana
Source: Front Immunol. 2025 Apr 9;16:1479689. doi: 10.3389/fimmu.2025.1479689 (PMC12014679; doi:10.3389/fimmu.2025.1479689)
Supplement: Supplementary file 3 [file Table1.docx]

|  | **D1** | | | | | | | **D2** | | | | | | | **D3** | | | | |
| --- | --- | --- | --- | --- | --- | --- | --- | --- | --- | --- | --- | --- | --- | --- | --- | --- | --- | --- | --- |
| **mouse** | 1 | 2 | 3 | 4 | 5 | 6 | 7 | 8 | 9 | 10 | 11 | 12 | 13 | 14 | 15 | 16 | 17 | 18 | 19 |
| **%**  **Hu-CD19+** | 0.05 | 0.01 | 0.02 | 0.12 | 0.01 | 0.06 | 0.11 | 0.03 | 0.12 | 0.08 | 0.09 | 0 | 0.05 | 0.06 | 0.16 | 0.01 | 0.05 | 0.07 | 0.04 |
| **%**  **Hu-CD14+** | 0.29 | 0.04 | 0.06 | 0.34 | 0 | 0.17 | 0.02 | 0.09 | 0 | 0.02 | 0.46 | 0.23 | 0.4 | 0.23 | 0.04 | 0.3 | 0.11 | 0.17 | 0.08 |

**Table S1**: Flow cytometric detection of human CD19^+^ and CD14^+^ cells in NSG mice. To detect relevant human cells, we excluded murine host cells by first gating anti-mouse Ly-6G/Ly-6C Pacific Blue (Gr-1). At least 50 000 events were acquired and analyzed using BD FACSDiva software v.6.1.2. The data shown are the percentages of markers from all mice transplanted with PBMCs from the three different donors (D1, D2, and D3).
