# Supplementary material for: Humanized mouse model reveals the immunogenicity of Hepatitis B Virus vaccine candidates produced in CRISPR/Cas9-edited Nicotiana benthamiana
Source: Front Immunol. 2025 Apr 9;16:1479689. doi: 10.3389/fimmu.2025.1479689 (PMC12014679; doi:10.3389/fimmu.2025.1479689)
Supplement: Supplementary file 4 [file Table2.docx]

Table S2. hIgG and hIgM titers for each individual mouse

| Mouse  ID | Donor  ID | Immunization  Group | hIgM (Endpoint Titer) | hIgG (Endpoint Titer) |
| --- | --- | --- | --- | --- |
|  |  |  | 35 days | 35 days |
| 1 | D1 | S/preS1^16-42^ | - | - |
| 2 |  |  | 13599.72 | 16557.08 |
| 3 |  |  | - | - |
| 4 |  |  | 31337.65 | 46195.49 |
| 5 | D1 | Addavax | 42635.96 | 37251.03 |
| 6 |  |  | - | - |
| 7 |  |  | - | - |
| 8 | D2 | S/preS1^16-42^ | 493.61 | 2294.03 |
| 9 |  |  | 17470.49 | 13025.34 |
| 10 |  |  | 4214.49 | 2392.45 |
| 11 |  |  | 831.91 | 2245.85 |
| 12 | D2 | Addavax | - | - |
| 13 |  |  | 4797.35 | 5165.32 |
| 14 |  |  | 2759.41 | 2785.77 |
| 15 | D3 | S/preS1^16-42^ | 23637.19 | 9486.01 |
| 16 |  |  | - | - |
| 17 |  |  | 7706.88 | 73032.89 |
| 18 | D3 | Addavax | - | - |
| 19 |  |  | 6837.58 | 4398.28 |
| “-” Mice showing clinical deterioration before day 35 were removed from the study.  hIgM and hIgG endpoint titers at 35-days post-immunization were calculated based on a 4-parameter logistic regression curve fitted to a pool of immune sera, as the reciprocal sample dilution that would result in three times baseline ± standard error as derived from the internal standard curve. | | | | |
